# Supplementary material for: miR-146a-5p mediates inflammation-induced β cell mitochondrial dysfunction and apoptosis
Source: J Biol Chem. 2024 Sep 27;300(11):107827. doi: 10.1016/j.jbc.2024.107827 (PMC11538863; doi:10.1016/j.jbc.2024.107827)
Supplement: Supplementary Figure Legends [file mmc1.docx]

**Supplementary Figure Legends**

**Figure S1. Pro-inflammatory stress increases miR-146a-5p expression in MIN6 stable cells and overexpression leads to cell death.** (A-C) Fold change values from qRT-PCR experiments (n=6) to determine expression level of miR-146a-5p in Scr and Scr+Cy MIN6 cells (A), ZIP and ZIP+Cy MIN6 cells (B), and OE and OE+Cy MIN6 cells (C). (D-F) Incucyte live cell image system to track cell death (represented by green or red fluorescence) over time. Cell death was compared between WT and Scr (D), WT and ZIP (E), and WT and OE (F) treated with or without cytokines. Scale = 400 µm. Unpaired t-test was used for comparing two groups. For all comparisons, *p*-values are indicated above.

**Figure S2. Overexpression of miR-146a-5p increases cleaved caspase-3 levels under pro-inflammatory stress.** (A) Western blot image showing abundance of caspase-3 and cleaved caspase-3 proteins in MIN6 cells (n=4) treated with/without pro-inflammatory cytokines. (B) Quantification of Western blots. Data is presented as means ± SEM and statistical significance was determined by one way ANOVA. For all comparisons, *p*-values are indicated above.
